# Predictors of failure of conservative treatment among patients with emphysematous pyelonephritis

**DOI:** 10.1186/1471-2334-14-418

**Published:** 2014-07-29

**Authors:** Yu-Chuan Lu, Bing-Juin Chiang, Yuan-Hung Pong, Kuo-How Huang, Po-Ren Hsueh, Chao-Yuan Huang, Yeong-Shiau Pu

**Affiliations:** Department of Urology, National Taiwan University Hospital, National Taiwan University, College of Medicine, 10002 No. 7 Chung-Shan South Road, Taipei, Taiwan; Departments of Laboratory Medicine and Internal Medicine, National Taiwan University Hospital, National Taiwan University College of Medicine, Taipei, Taiwan

**Keywords:** Emphysematous pyelonephritis, Percutaneous drainage, Conservative management, Severe hypoalbuminemia, Empirical antibiotic regimen

## Abstract

**Background:**

Emphysematous pyelonephritis (EPN) is a severe necrotizing infection of the renal parenchyma and perirenal tissues that is caused by gas-producing bacterial pathogens. Percutaneous drainage is now the gold standard of definitive management. The aim of this study is to analyze the predictors associated with failure of conservative treatment among patients with EPN and offer the recommendation of appropriate empirical antibiotic regimen.

**Methods:**

From January 2001 to December 2013, 44 consecutive patients were diagnosed with EPN. The demographic characteristics, clinical presentations, management strategies, and final outcomes were analyzed retrospectively.

**Results:**

The overall survival rate was 88.6% (39/44). Need for emergency hemodialysis, shock on initial presentation, altered mental status, severe hypoalbuminemia, inappropriate empirical antibiotic treatment and polymicrobial infections were significantly more common in the patients who died compared with the survivors. The overall failure rate of conservative treatment was 32.6% (14/43). Severe hypoalbuminemia (*p* = 0.003), need for emergency hemodialysis (*p* = 0.03), and polymicrobial infections (*p* = 0.04) were significantly associated with failure of conservative treatment. Severe hypoalbuminemia was independently associated with conservative management failure (*p* = 0.02). Even in the patients treated with percutaneous drainage plus effective antibiotics, failure was still associated with severe hypoalbuminemia (*p* = 0.01). According to the in vitro susceptibility data, third-generation cephalosporins is recommended as the empirical antibiotic regimen.

**Conclusions:**

Both appropriate empirical antibiotic and percutaneous drainage were essential for patients with EPN. Patients with severe hypoalbuminemia had a higher risk of conservative treatment failure, and additional management may be required.

**Electronic supplementary material:**

The online version of this article (doi:10.1186/1471-2334-14-418) contains supplementary material, which is available to authorized users.

## Background

Emphysematous pyelonephritis (EPN) is a severe necrotizing infection of the kidneys that is characterized by bacterial production of gas within the renal parenchyma [1]. It occurs most commonly in female diabetic patients (70% to 90%), and has a mortality rate of up to 80% when patients are only treated medically [1, 2].

Treatment options for EPN have evolved over the years, from invasive surgery to more conservative approaches including percutaneous catheter drainage (PCD) or the use of a double-J catheter (DBJ). Until the late 1980s, the management of EPN usually involved emergency nephrectomy and/or open surgical drainage with antibiotic treatment [1], however this approach was associated with a mortality rate of 40% to 50% [3]. The implementation of PCD techniques facilitates maximum nephron sparing and restoration of renal function [4]. Over the last two decades, improvements in management techniques have resulted in a decrease in the mortality rate to 21% [5].

Percutaneous drainage, rather than nephrectomy, is now the gold standard of care and definitive management for the majority of patients with EPN [6]. This strategy is associated with a lower mortality rate than medical management or emergency nephrectomy. Even though conservative treatment is favorable, salvage nephrectomy or open surgical drainage is still necessary in a small proportion of patients. However, there is limited information about the usefulness of clinical parameters to classify patients who are likely to fail conservative treatment. This retrospective study aimed to determine the factors that may predict the failure of conservative treatment in patients with EPN.

## Methods

This study was approved by the Institutional Review Board and Ethics Committee of National Taiwan University Hospital. Forty-four consecutive patients with EPN were treated at the National Taiwan University Hospital from January 2001 to December 2013. Information on clinical and demographic profiles including age, sex, underlying medical conditions, laboratory investigations, clinical features at initial presentation, imaging studies, type of management, and outcomes of the patients were obtained from medical charts. The clinical features included signs and symptoms at presentation, and the patients’ hemodynamic and mental status. The laboratory variables included white blood cell count, platelet count, C-reactive protein, serum albumin, serum sodium, hemoglobin A1C (HbA1C), and serum creatinine, as well as the results of urinalysis and blood, wound, and urine cultures, mostly obtained at the initial presentation. The initial management modalities for EPN consisted of antibiotics alone, PCD with antibiotics, an indwelling double-J catheter with antibiotics and emergency nephrectomy. Conservative treatment of EPN comprised antibiotics alone and PCD or an indwelling double-J catheter with antibiotics. Appropriate empirical antibiotic treatment had to be treatment matching the in vitro susceptibility of the pathogens.

### Definitions

According to the classification system of Huang and Tseng [7], which is based on the extent of air seen on computed tomography (CT), the patients were divided into one of the following five EPN classifications: class 1, gas in the collecting system only; class 2, gas in the renal parenchyma without extension to the extrarenal space; class 3A, extension of gas or abscess to the perinephric space; class 3B, extension of gas or abscess to the pararenal space; and class 4, bilateral EPN or a solitary kidney with EPN. Thrombocytopenia was defined as a platelet count less than 120,000/mL, severe hypoalbuminemia as serum albumin < 3.0 g/dL, and hyponatremia as serum sodium < 135 mEq/L. Shock was defined as a systolic pressure less than 90 mmHg and evidence of end organ damage including the respiratory system, liver, or kidneys. The patients with an absolute increase in serum creatinine of ≥ 0.3 mg/dL after admission compared with baseline were diagnosed with acute kidney injury. Recurrent EPN was diagnosed when both a clinical presentation of sepsis and progressive lesions on imaging studies were noted within three months after adequate treatment for EPN.

We defined success of conservative treatment as improvement in clinical condition, decrement of air distribution in follow-up images, and discharge alive after PCD, DBJ indwelling or antibiotics alone. Failure of conservative treatment was defined as intra-hospital mortality, recurrent EPN, or appearance of unstable hemodynamics or a prolonged fever after conservative treatment within seven days. Salvage nephrectomy or open drainage was performed if conservative treatment failed.

### Statistical analysis

Categorical variables were compared using the chi-square test or Fisher’s exact test. Continuous variables were expressed as median values, and the Wilcoxon rank sum test was used for comparisons. The independent variables with a *p-*value ≤ 0.05 in the univariate analysis were selected for logistic regression analysis to identify the independent factors significantly associated with occurrence of conservative treatment failure. A *p-*value of ≤ 0.05 was considered to be statistically significant.

## Results

**Figure 1:**
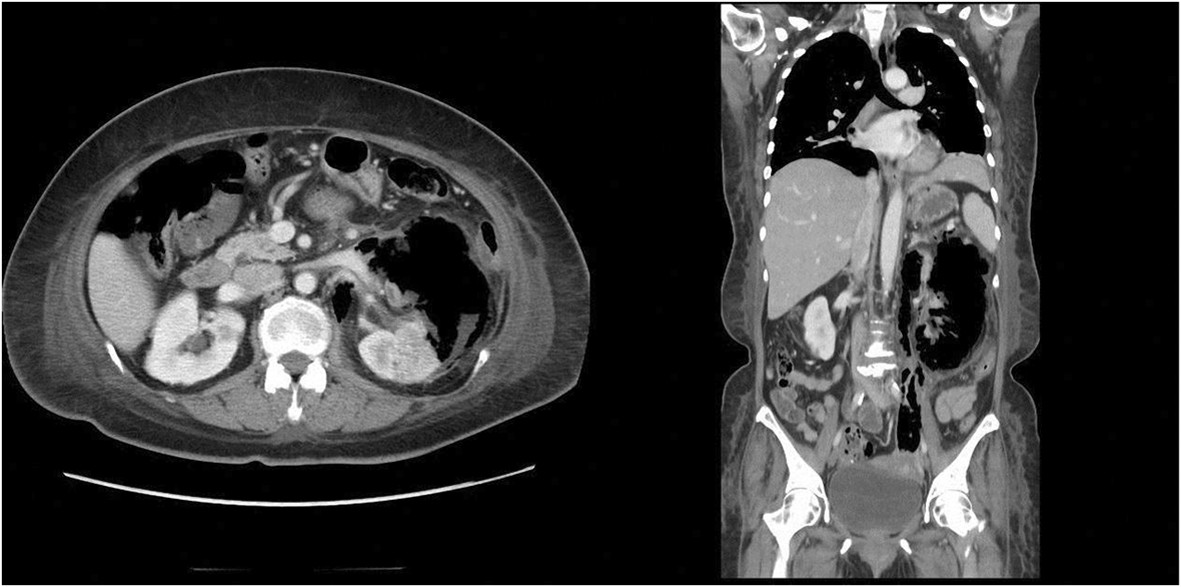
**Contrast CT revealed the presence of gas in the left pelvicalyceal system with extending into paranephric area (Class 3B).**

The mean (standard deviation) age of the patients was 59.9 (15.5) years. There were 32 women (72.7%) and 12 men. Thirty patients (68.2%) had type II diabetes mellitus, and obstructive uropathy was the second most common predisposing factor (23 patients, 52.3%). Urolithiasis occurred in half of the patients. Other comorbidities included hypertension (17 patients, 38.6%), cerebrovascular accidents (5 patients, 11.4%), liver cirrhosis (4 patients, 9.1%), Down syndrome (1 patient, 2.3%), and acquired immunodeficiency syndrome (1 patient, 2.3%). The left kidney was more frequently affected than the right (28 vs. 18); 2 patient had bilateral involvement and 2 had EPN of a solitary kidney. Table 1 demonstrates the clinical and epidemiological characteristics of the surviving and non-surviving patients.Shock occurred in 9 patients, and 15 patients had altered mental status upon initial presentation. Acute kidney injury was noted in 22 patients. Five patients underwent emergency hemodialysis, 3 of whom died. The mean leukocyte count at admission was 15286/μL. Thrombocytopenia and hypoalbuminemia were present in 11 (25%) and 22 (50%) patients, respectively. Eight patients had class 1 EPN, 12 had class 2, 11 had class 3A, 7 had class 3B (Figure 1), and 4 had class 4 based on CT imaging findings; CT data were missing for 2 patients.

**Table 1:** **Demographic data and clinical characteristics of the patients with emphysematous pyelonephritis**

Variable, n (%)	Surviving (n = 39)	Died (n = 5)	*P*-value	OR	*P*-value
Median age (y)	60	65	0.32	1. (0.9-1.1)	0.40
Female sex	27 (69.2)	5 (100)	0.30	-	-
**Underlying condition**					
Diabetes mellitus	27 (69.2)	3 (60)	0.65	0.7 (0.1-4.5)	0.68
Hypertension	14 (35.9)	3 (60)	0.36	2.7 (0.4-18)	0.31
Prior cerebrovascular accident	4 (10.3)	1 (20)	0.47	2.2 (0.2-24.7)	0.53
Down syndrome	1 (2.6)	0 (0)	1.00	-	-
Liver cirrhosis	3 (7.7)	1 (20)	0.39	3 (0.3-36.1)	0.39
Obstructive uropathy	19 (48.7)	4 (80)	0.35	4.2 (0.4-41.1)	0.22
Urolithasis	19 (48.7)	3 (60)	1.00	1.6 (0.2-10.5)	0.64
**Laboratory finding**					
Leukocyte (10^3^/μL)	13.8	15.40	0.77	1.0 (0.9-1.1)	0.83
Platelet count (10^3^/mL)	195.0	106.0	0.08	0.9 (0.8-1.0)	0.15
Thrombocytopenia	8 (20.5)	3 (60)	0.09	5.8 (0.8-40.9)	0.08
(≤120,000/mL)					
Albumin (g/dL)	2.7	2.8	0.43	0.4 (0.1-2.5)	0.36
Hypoalbuminemia	17 (47.2)	5 (100)	0.05	-	-
(<3.0 g/dL)					
Hyponatremia	10 (25.6)	2 (40)	0.60	1.9 (0.3-13.3)	0.50
C-reactive Protein (mg/dL)	9.2	5.98	0.67	1.0 (0.9-1.1)	0.68
Pyuria	32 (88.9)	5 (100)	1.00	-	-
Hematuria	21 (58.3)	5 (100)	0.14	-	-
HbA1C >8%	17 (47.2)	2 (40)	1.00	0.7 (0.1-5.6)	0.73
**Image study**					
Radiologic classification	21 (56.8)	1 (20)	0.17	0.2 (0.02-2.3)	0.22
(3A,3B,4)^a^					
Involved site (left)	23 (59.0)	3 (60)	1.00	0.9 (0.1-6.3)	0.95
Bilateral involvement	2 (5.1)	0 (0)	1.00	-	-
Need for hemodialysis	2 (5.1)	3 (60)	0.01	27.8 (2.8-272.9)	0.004
Inappropriate empirical antibiotic use	3 (8.6)	3 (60)	0.02	16.0 (1.9-136.7)	0.01
Bacteremia	16 (41.0)	4 (80)	0.16	5.8 (0.6-56.4)	0.13
Shock	5 (12.8)	4 (80)	0.004	27.2 (2.5-295.0)	0.01
Acute kidney injury	18 (46.2)	4 (80)	0.35	4.7 (0.5-45.6)	0.19
Altered mental status	11 (28.2)	4 (80)	0.04	10.2 (1.0-101.5)	0.05
Polymicrobial infection	7 (18.0)	5 (100)	0.001	-	-

^a^Radiologic classification is based on the extent of air seen on computed tomography (CT): class 3A, extension of gas or abscess to the perinephric space; class 3B, extension of gas or abscess to the pararenal space; class 4, bilateral EPN or a solitary kidney with EPN.

Third-generation cephalosporin (18/44, 40.9%) were most often prescribed initially to the patients. Antibiotics were adjusted after available blood, urine or pus cultures. *Escherichia coli* was the most common causative organism cultured from urine, blood or wound specimens, present in 21 (47.7%) patients. The other organisms isolated included *Proteus mirabilis* (20.5%), *Klebsiella pneumonia*e (15.9%), *Enterococcus* species (15.9%), *Pseudomonas aeruginosa* (11.4%), and *Candida* species (11.4%). Polymicrobial infections were found in 12 patients (27.3%). Urine cultures were positive in 33 patients, and 19 patients had *E. coli* infections. Bacteremia occurred in 20 patients (43.9%), with *E. coli* bacteremia in 12 patients. Cultures of wound pus were positive in 21 patients, with *E. coli* in 10, *P. mirabilis* in 7, and *K. pneumonia*e in 3 (Table 2). Table 3 showed the in vitro susceptibility of the causative pathogens.

**Table 2:** **Causative organisms from blood, urine and wound/pus**

Urine culture (33 cases, 75.0%)	Wound/Pus culture (21 cases, 47.7%)	Blood culture (20 cases, 45.5%)	Overall
*E. coli* (19)	*E. coli* (10)	*E. coli* (12)	*E. coli* (21)
Polymicrobial (11)	*P. mirabilis* (7)	*K. pneumonia*e (3)	*P. mirabilis* (9)
	*K. pneumonia*e (3)	Polymicrobial (1)	*Enterococcus* species (7)
	Polymicrobial (3)		*K. pneumonia*e (7)
			*P. aeruginosa* (5)
			*Candida* species (5)
			Polymicrobial (12)

**Table 3:** **Susceptibility rate of causative organisms to antibiotics**

	*E. coli*(n = 21)	*P. mirabilis*(n = 9)	*K. pneumoniae*(n = 7)	*Enterococcus*species (n = 7)	*P. aeruginosa*(n = 5)
Antibiotics			% Susceptible		
Ampicillin	25	33.3	0	40	-
Amoxicillin	65	100	40	-	-
Amikacin	100	100	100	-	80
Cefazolin	64.7	83.3	20	-	-
Cefuroxime	75	100	0	-	-
Cefotaxime	90	100	60	-	-
Cefmetazole	88.9	100	60	-	-
Ceftazidime	100	100	100	-	80
Ciprofloxacin	70.6	100	50	20	80
Levofloxacin	70	100	50	-	80
Piperacillin/tazobactam	88.2	100	75	-	75
Cefepime	95	100	100	-	60
Imipenem	100	100	100	-	100
Ertapenem	100	100	100	-	
Meropenem	100	-	-	-	66.7
Cotrimoxazole	38.5	40	50	-	
Gentamicin	70	66.7	100	-	25
Tetracycline	-	-	-	40	-
Teicoplanin	-	-	-	60	-
Vancomycin	-	-	-	100	-
Linezolid	-	-	-	100	-
Aztreonam	-	-	-	0	80

The overall survival rate was 88.6% (39/44). Need for emergency hemodialysis (*p* = 0.01), shock on initial presentation (*p* = 0.004), altered mental status (*p* = 0.04), severe hypoalbuminemia (*p* = 0.05), and inappropriate empirical antibiotic treatment (*p* = 0.02) and polymicrobial infections (*p* = 0.001) were significantly more common in the non-survivors than in the survivors. There were no significant differences between the non-survivors and survivors with regard to age, history of diabetes mellitus, thrombocytopenia, acute kidney injury, serum creatinine level, leukocyte count, presence of microscopic hematuria, pyuria, urinary tract obstruction, or urolithiasis.

**Figure 2:**
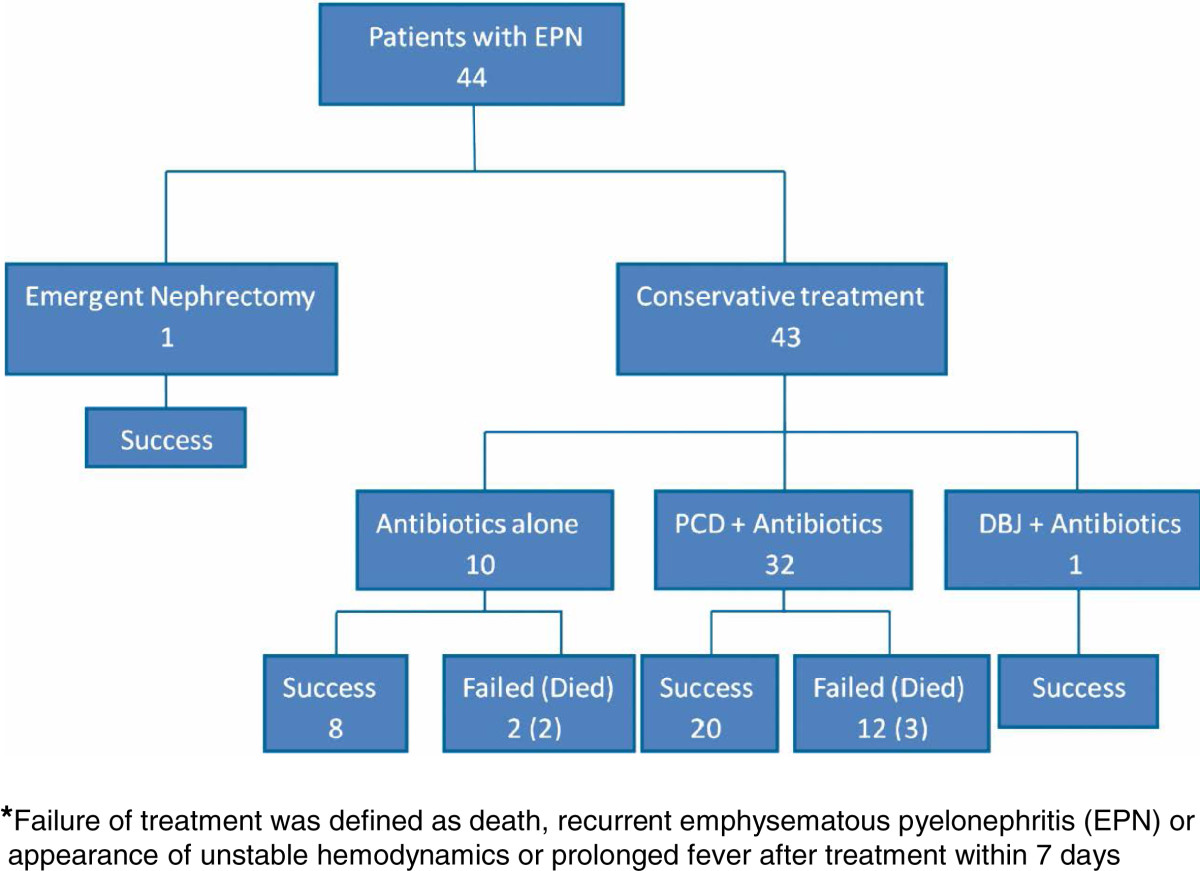
**Study cohort by outcome.** Values indicate the number of patients.

Ten patients received antibiotics alone, and treatment was successful in 80.0% of these patients (2 patients died). Third-generation cephalosporins were the most frequently used antibiotics and were used in 7 patients (for cases caused by E. coli or P. mirabilis). One patient underwent emergency nephrectomy and survived. One patient underwent ureteroscopy with double-J catheter stenting and survived. Thirty-two patients were treated with PCD and antibiotics, 20 of whom did well (Figure 2). However, 8 patients experienced recurrent EPN within 3 months, and 3 patients died thereafter. Persistent fever was noted in 4 patients after PCD. Overall, 5 patients received salvage nephrectomy, 1 underwent open drainage and 1 required repeated PCD. Elective nephrectomy was carried out in 2 patients with poorly functioning kidneys. The overall failure rate of conservative therapy was 32.6% (14/43). Severe hypoalbuminemia (p = 0.003), need for emergency hemodialysis (p = 0.03), and polymicrobial infections (p = 0.04) were significantly associated with failure of conservative treatment (Table 4). Table 5 shows the results of multivariate analysis of the three significant variables, in which severe hypoalbuminemia was independently associated with failure of conservative therapy (p = 0.02).

**Table 4:** **Factors associated with failure of conservative treatment**

Variable, n (%)	Success group (n = 29)	Failure group (n = 14)	*P*-value	OR	*P*-value
Hypoalbuminemia	9 (34.6)	12 (85.7)	0.003	11.3 (2.1-62.1)	0.01
(<3.0 g/dL)					
Need for hemodialysis	1 (3.5)	4 (28.6)	0.03	11.2 (1.1-112.5)	0.04
Polymicrobial infection	5 (17.2)	7 (50)	0.04	4.8 (1.2-20.0)	0.03

**Table 5:** **Multivariate analysis of independent risk factors for failure of conservative treatment**

Variable	OR	*P*-value
Need for hemodialysis	2.8 (0.2-35.5)	0.42
Severe hypoalbuminemia	7.9 (1.3-46.4)	0.02
(<3.0 g/dL)		
Polymicrobial infection	2.2 (0.4-12.1)	0.38

Among the 32 patients who received PCD, treatment failed in 12. Only severe hypoalbuminemia had association with PCD failure (*p* = 0.01). The patients with hypoalbuminemia had a significantly higher incidence of conservative therapy failure (57.1%) and PCD failure (55.6%).

## Discussion

A high level of awareness is required for patients with EPN due to the life-threatening nature of the associated septic complications. Treatment of EPN initially involves fluid and electrolyte resuscitation, antibiotic therapy, glycemic control, and relief of urinary tract obstruction where appropriate. Further management consists of either conservative medical treatment with antibiotics, emergency nephrectomy with medical therapy, or PCD with medical therapy. The treatment of EPN has evolved over the years from invasive surgery to more conservative approaches.

Some studies advocate nephrectomy for patients with EPN, in combination with antibiotics, fluids, and optimization of blood glucose and electrolytes [3, 8]. However, the mortality rate in a series by Ahlering et al. advocating emergency nephrectomy was 42% [3]. Some specialists have proposed that conservative treatment or PCD should only be used for select cases such as localized EPN, bilateral disease, EPN in a solitary kidney, or if the patient cannot tolerate general anesthesia [9]. Medical treatment with antibiotics alone carries the highest risk of mortality [1]. However, in the current study, 10 patients received antibiotics alone, and the mortality rate was 20% (2 patients died). Most of the cases in the current study were limited EPN (class 1 & 2) and were comparatively healthy. Six patients had class 1 EPN, 3 had class 2, and 1 had class 3A with a small air pocket. This may account for the superior outcomes compared with prior studies. Thirty-two patients underwent PCD plus antibiotics, and 3 patients (9.4%) eventually died. In a meta-analysis of management strategies, the mortality rate with medical treatment alone was 50%, and for PCD plus medical treatment the mortality rate was 13.5% [5]. The reported mortality rate with PCD is consistent with our results. However, in our study, 8 patients experienced recurrent EPN, and 4 patients had persistent fever after PCD. The failure rate of PCD was 37.5%.

We then investigated the factors that were predictive of mortality and failure of conservative treatment. To the best of our knowledge, this is the first study to specifically analyze the predictors associated with conservative management failure of EPN and offer the recommendation of appropriate empirical antibiotic. It is also one of the largest studies investigating the prognostic factors of EPN.

Diabetes mellitus appeared to be the most common risk factor for developing EPN. It is believed that a high tissue glucose concentration may provide a favorable environment for the growth of gas-producing bacteria and for the inhibition of leukocyte function, which then impairs the response to infection [10]. Nevertheless, diabetes was not found to be associated with increased mortality (*p* = 0.65, odds ratio [OR] = 0.67) or with failure of conservative treatment (*p* = 1.00, OR = 1.32) in the present study, even among the patients with poorly controlled diabetes (HbA1C > 8%). Similarly, there were no significant associations between a higher mortality rate in the patients with EPN and sex, age, or other comorbidities such as liver cirrhosis, hypertension, and history of cerebrovascular accidents.

Serum albumin is the most abundant protein in human blood plasma and constitutes approximately half of the blood serum proteins. Albumin transports hormones, fatty acids, and other compounds, buffers blood pH, and maintains osmotic pressure. Albumin synthesis is suppressed in response to inflammatory conditions and malnutrition [11]. In sepsis, infection, trauma, or major surgery, albumin level decreases by 1–1.5 g/dl over 3–7 days. In a study of over 15,000 severely ill patients, an admission serum albumin < 3.4 g/dl has been correlated with increased of mortality [12]. Hypoalbuminemia was found to be a strong predictor of increased mortality and morbidity in surgical or ICU patients [13]. It has been reported that survival after acute kidney injury is significantly altered according to the level of serum albumin [14]. Accelerated protein breakdown is a feature of the metabolic alterations seen in patients with acute kidney injury. In our previous study, severe hypoalbuminemia (<3.0 g/dL) combined with acute kidney injury was associated with an increased mortality rate (42.9%, *p* = 0.03) in patients with EPN. In the present study, severe hypoalbuminemia was independently associated with an increase in the failure of conservative treatment. Even in the patients treated with PCD plus antibiotics, the failure rate was still independently associated with severe hypoalbuminemia. Therefore, the patients with severe hypoalbuminemia had a higher risk of conservative treatment failure and additional management including salvage nephrectomy, open drainage or repeated PCD was required. Hypoalbuminemia is probably related to infection, however, it can also be the result of a previous illness, such as liver cirrhosis. When the patients with liver cirrhosis was excluded, there was still association between PCD failure and severe hypoalbuminemia (n = 30, *p* = 0.01). It is not known whether albumin concentration itself is important or whether it is simply a marker of poor general health. The benefit of correcting hypoalbuminemia in this situation has not been clearly established. However, PCD with antibiotics is still recommended in the initial stage for the patients who have severe hypoalbuminemia, as it immediately reduces renal tissue pressure and decreases the mortality rate.

In a literature review, many prognostic factors for mortality were identified, however none of the trials studied a large population. Khaira et al. [15] also reported that shock was an independent poor prognostic risk factor in a case series of 19 patients with EPN. Huang and Tseng [7] reported that thrombocytopenia, altered mental status, severe proteinuria, and acute renal failure at the presentation of EPN were associated with a poor outcome. Similarly, a study conducted in India of 39 patients with EPN showed that altered mental status, thrombocytopenia, renal failure, and severe hyponatremia at presentation were also associated with higher mortality rates [16]. In a meta-analysis, systolic blood pressure less than 90 mmHg, serum creatinine greater than 2.5 mg/dL, and impairment of consciousness were also found to be associated with increased mortality [17]. In the present study of 44 patients with EPN, need for emergency hemodialysis, shock on initial presentation, altered mental status, severe hypoalbuminemia, inappropriate empirical antibiotic regimen and polymicrobial infections were significantly associated with mortality. With regards to the classification of EPN based on the degree of gas seen on CT, the outcomes of the patients with class 3 and 4 EPN were not statistically different from those with class 1 and 2 disease, which is in agreement with previously reported studies [15]. Even though Huang and Tseng reported a tendency towards higher mortality and failure rates with PCD in extensive disease (class 3 and 4) [7], we did not find this association. The alternative approach of conservative treatment also had no association with the failure rate (*p* = 0.23).

Microbial organisms cultured from the urine, kidneys and blood of patients with EPN include the common urinary pathogens, *E. coli*, *K. pneumoniae* and *P. mirabilis*. In the present study, other organisms cultured consist of *P. aeruginosa* and *Enterococcus* species. Infection by anaerobic organisms (for example *Bacteroides fragilis* and *Clostridium septicum*) has also been reported [18, 19]. It is common wisdom that appropriate empirical antibiotic treatment (i.e., matching the in vitro susceptibilities of the isolated pathogens) reduces mortality. In EPN patient, we also found that inadequate empirical antibiotic use was significantly associated with mortality. The choice of empirical antibiotic regimen should be appropriate for targeting Gram negative bacteria. Third-generation cephalosporins, for example ceftazidime, is recommended as the initial antibiotic regimen because of high susceptibility and anti-pseudomonal activity. In patients with severe sepsis and septic shock, vancomycin plus imipenem or ceftazidime as the empirical choice is suggested. However, fluoroquinolone should be avoided because of lower susceptibility rate among common gram-negative bacteria in Taiwan [20]. Approximately 70% of the *E. coli* isolates and 50% *K. pneumoniae* isolates were susceptible to fluoroquinolones.

This study has limitations inherent to all retrospective, single institution studies. The small number of patients may account for the lack of significance of some of the factors analyzed. Second, we mostly used data acquired at the initial presentation to identify the risk factors for mortality. Factors that varied over the time of treatment may also have influenced outcomes. A larger prospective cohort study is required to support our findings.

## Conclusions

Severe hypoalbuminemia was associated with the failure of conservative management and PCD. Patients with severe hypoalbuminemia had a higher risk of conservative treatment failure, and additional management may be required for these patients.

## Authors’ original submitted files for images

Below are the links to the authors’ original submitted files for images. Authors’ original file for figure 1 Authors’ original file for figure 2

## Abbreviations

EPN: Emphysematous pyelonephritis
PCD: Percutaneous catheter drainage
DBJ: Double-J catheter
HbA1c: Hemoglobin A1c
CT: Computed tomography
SD: Standard deviation
OR: Odds ratio.
