# Supplementary figures and images for: Predictors of failure of conservative treatment among patients with emphysematous pyelonephritis
Source: BMC Infect Dis. 2014 Jul 29;14:418. doi: 10.1186/1471-2334-14-418 (PMC4124134; doi:10.1186/1471-2334-14-418)

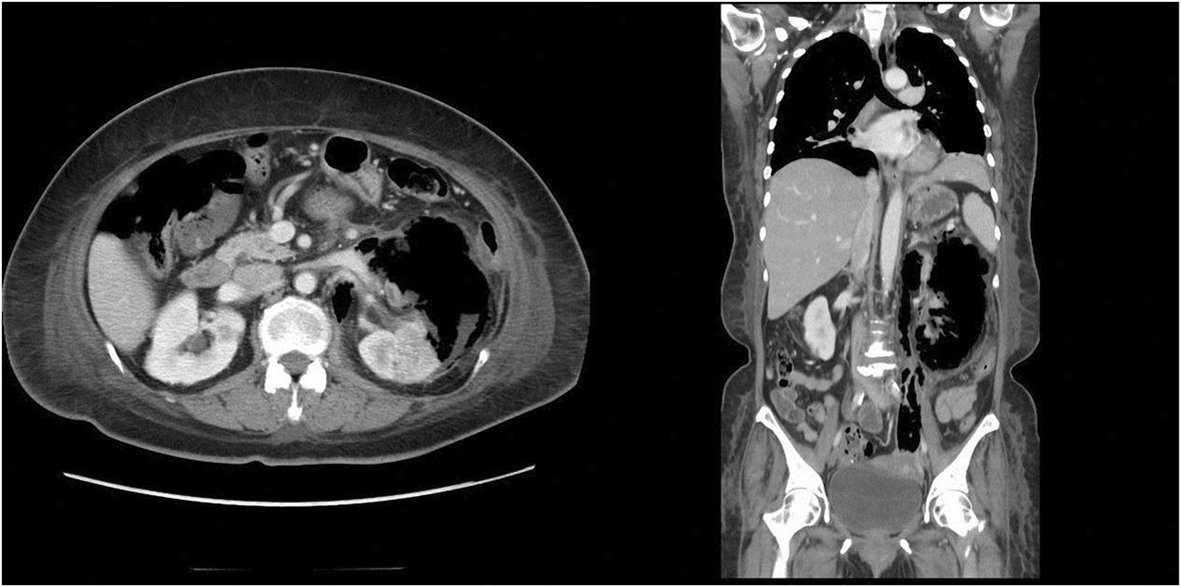

Supplement: Supplementary file 1 — Authors’ original file for figure 1 [file 12879_2014_3722_MOESM1_ESM.tif]

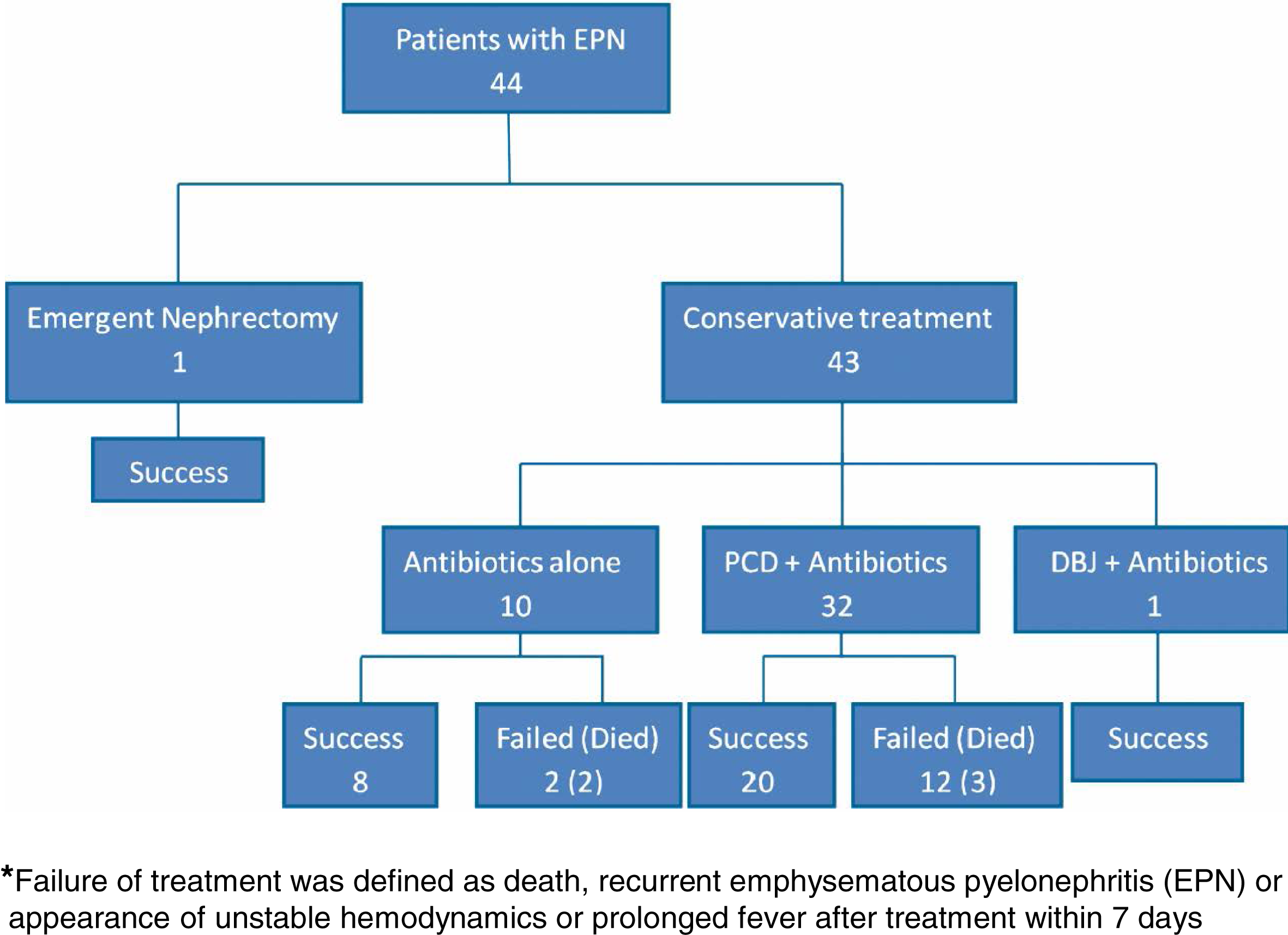

Supplement: Supplementary file 2 — Authors’ original file for figure 2 [file 12879_2014_3722_MOESM2_ESM.tif]
